# Spike‐Based Neuromorphic Hardware for Dynamic Tactile Perception with a Self‐Powered Mechanoreceptor Array

**DOI:** 10.1002/advs.202402175

**Published:** 2024-07-09

**Authors:** Sang‐Won Lee, Seong‐Yun Yun, Joon‐Kyu Han, Young‐Hoon Nho, Seung‐Bae Jeon, Yang‐Kyu Choi

**Affiliations:** ^1^ School of Electrical Engineering Korea Advanced Institute of Science and Technology (KAIST) 291 Daehak‐ro, Yuseong‐gu Daejeon 34141 Republic of Korea; ^2^ System Semiconductor Engineering and Department of Electronic Engineering Sogang University 35 Baekbeom‐ro, Mapo‐gu Seoul 04107 Republic of Korea; ^3^ Department of Neurosurgery University of Pennsylvania Philadelphia PA 19104 USA; ^4^ Department of Electronic Engineering Hanbat National University 125 Dongseo‐daero, Yuseong‐gu Daejeon 34158 Republic of Korea

**Keywords:** artificial mechanoreceptor array, biristor, dynamic gesture recognition, spiking neural network (SNN), triboelectric nanogenerator (TENG)

## Abstract

A self‐powered mechanoreceptor array is demonstrated using four mechanoreceptor cells for recognition of dynamic touch gestures. Each cell consists of a triboelectric nanogenerator (TENG) for touch sensing and a bi‐stable resistor (biristor) for spike encoding. It produces informative spike signals by sensing a force of an external touch and encoding the force into the number of spikes. An array of the mechanoreceptor cells is utilized to monitor various touch gestures and it successfully generated spike signals corresponding to all the gestures. To validate the practicality of the mechanoreceptor array, a spiking neural network (SNN), highly attractive for power consumption compared to the conventional von Neumann architecture, is used for the identification of touch gestures. The measured spiking signals are reflected as inputs for the SNN simulations. Consequently, touch gestures are classified with a high accuracy rate of 92.5%. The proposed mechanoreceptor array emerges as a promising candidate for a building block of tactile in‐sensor computing in the era of the Internet of Things (IoT), due to the low cost and high manufacturability of the TENG. This eliminates the need for a power supply, coupled with the intrinsic high throughput of the Si‐based biristor employing complementary metal–oxide–semiconductor (CMOS) technology.

## Graphical abstract

A self‐powered artificial mechanoreceptor array is demonstrated by combining triboelectric nanogenerators and bi‐stable resistors. The artificial mechanoreceptor encodes information with spike signals, thus allowing a spike‐based neuromorphic computing architecture. The mechanoreceptor array monitors spatiotemporal touch gestures with high force resolution in both the spatial and temporal domains. Dynamic tactile perception with the help of a spiking neural network is demonstrated.

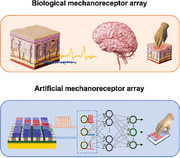

## 1 Introduction

A biological sensory system can detect various signals from the environment and provide feedback with informative data by appropriately processing these signals.^[^ ^1^ ^]^ Among these systems, the biological tactile system discerns physical contact events based on signals received from mechanoreceptors in the skin.^[^ ^2^, ^3^, ^4^ ^]^ With the aid of dispersed mechanoreceptors throughout the human body, the tactile system acts as a sensor network, composed of highly distributed sensors, and has the capability of perceiving intricate patterns, various objects and hazardous situations. This capability is highly desired in various fields as well, such as robotics, prosthetics, diagnosis, and human‐machine interfaces; therefore, hardware implementation of a tactile perception system with a touch sensor array has strongly attracted the attention of researchers in this field.^[^ ^5^, ^6^, ^7^, ^8^, ^9^ ^]^ In order to realize such a tactile perception system with conventional computing architecture (Figure S1, Supporting Information), two bottlenecks which increase the overall power consumption and cost are unavoidable. First, the system requires an analog‐to‐digital converter (ADC) to transduce the analog signals from the touch sensor array into digital signals. This component corresponds to the first bottleneck of overall tactile perception system with the conventional architecture. In addition, the digital output from the ADC is transmitted to a central processing unit (CPU) and a memory unit. For high accuracy of perception, a proper training process should be conducted. During the training process, a considerable volume of data must shuttle between the CPU and the memory unit, driving up the operation time and increasing the power consumption as well. This is known as the von‐Neumann bottleneck, which corresponds to the second bottleneck of the overall system.^[^ ^10^ ^]^ The aforementioned bottlenecks limit the applicability of tactile perception modules for various computations in an edge system or an Internet of Things (IoT) system. To implement low‐power tactile perception modules, for instance, neuromorphic hardware inspired by biological brain was recently reported.^[^ ^11^, ^12^, ^13^ ^]^

**Figure 1:**
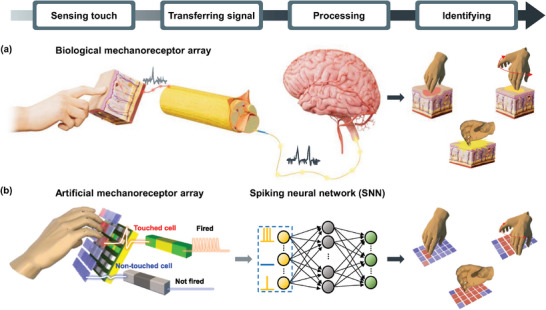
Comparison between a biological and the proposed artificial tactile system with a self‐powered artificial mechanoreceptor array. a) Schematic diagram of a biological tactile system based on mechanoreceptors in the human body. Biological mechanoreceptor cells distributed in the skin detect physical contact with a subtle pressure. Mechanoreceptor cells generate spikes and transmit them to a bundle of nerve fibers when mechanical pressure is sensed. These spikes are eventually delivered to the brain for perception, after which the brain classifies various touch gestures. b) Schematic diagram of the proposed mechanoreceptor array. Each mechanoreceptor cell in the array is composed of a TENG and a biristor. When external force is applied to the TENG, it generates current of *I*_TENG_, which is transferred to the biristor as *I*_in_. The biristor then produces a set of spikes, which can be applied to the SNN as input data for tactile perception.

There have been several attempts to eliminate such bottlenecks by mimicking the biological system even in terms of signal transmission and data processing.^[^ ^14^, ^15^, ^16^ ^]^ **Figure** **1**a illustrates the signal processing in the biological tactile system, where signals from biological mechanoreceptors are transmitted in the form of spikes. Due to this spike‐based characteristic, complicated tactile perceptions can be realized with extremely low energy. Also, a neural network composed of numerous neurons and synapses provides more energy‐efficient perception compared to computing hardware with the von‐Neumann architecture. Hence, a bio‐inspired artificial system capable of encoding spike signals according to the input tactile stimulus and realizing perception with a novel computing architecture, devoid of a data bus, represents a milestone in the development of a tactile sensory system with low power consumption for edge device applications.

To implement this system, an artificial mechanoreceptor module capable of detecting force and encoding spike signals is indispensable. To achieve this, two components are required: one for sensing and one for encoding. The first component is the sensing device, which detects external force and subsequently converts mechanical signals into electrical signals without the use of an extra transducer. The second component is the processing device, which encodes the spike signals by processing the electrical signals from the sensor without the use of an extra converter. In relation to this, an artificial mechanoreceptor module that combines a touch sensor with a spike‐generating device has been demonstrated.^[^ ^17^, ^18^, ^19^ ^]^ However, both components required an additional power supply. There have also been a few recent attempts to remove the power supply from an artificial mechanoreceptor module using a self‐powered touch sensor such as a piezoelectric nanogenerator (PENG) or a triboelectric nanogenerator (TENG).^[^ ^20^, ^21^, ^22^, ^23^ ^]^ As an example, one earlier report focused on primitive breath monitoring using the aforementioned mechanoreceptor.^[^ ^21^ ^]^ However, there is still a need for array‐level integration of artificial mechanoreceptors to perceive more complex gestures under a spatiotemporal and dynamic condition. On the other hand, numerous efforts have been made to enhance power and energy efficiency in energy harvesters. However, it is also important to explore proper applications, such as IoT systems, which can efficiently utilize small amounts of harvested energy. This approach shifts the focus from solely maximizing the harvested energy to maximizing its utility in self‐powered and self‐aware tiny systems.

On the other hand, reducing the power consumption required for signal processing during perception is crucial. This goal can be achieved using a spiking neural network (SNN) of the type widely employed for energy‐efficient systems.^[^ ^24^, ^25^ ^]^ As an alternative to the von‐Neumann architecture, the neuromorphic architecture, composed of an artificial spiking neuron and a synapse, has garnered considerable attention to address the aforementioned von‐Neumann bottleneck.^[^ ^26^ ^]^ This architecture is applicable to an artificial sensory module connected by sensory neurons and synapses for in‐sensor computing. Herein, the sensory neuron can sense a stimulus, generate a corresponding spike signal, and transmit it from the input layer to the output layer, while the synapse can memorize and update the weight values autonomously.

In this work, a self‐powered artificial mechanoreceptor array is demonstrated. Figure 1b shows the overall concept of the mechanoreceptor array. The mechanoreceptor cell is composed of a touch sensor and a bi‐stable resistor (biristor), which are serially connected. While the former serves as a touch sensor and an energy harvester removing power supply, the latter acts as a spike‐encoding device. When external force is applied to the TENG, the resulting current is transferred to the biristor as an input. Subsequently, the biristor produces multiple spike signals as an output. It should be noted that the TENG is considered to be a promising candidate for a mechanoreceptor module, offering advantages such as a low cost, high manufacturability, and good flexibility.^[^ ^27^, ^28^, ^29^ ^]^ Similarly, the biristor is an attractive candidate for an artificial neuron that receives current and converts it to a signal in the form of spikes. This is due to its small footprint, compatibility with complementary metal‐oxide‐semiconductor (CMOS) technology, cost‐effectiveness, and its capability for mass productivity and high throughput.^[^ ^30^, ^31^ ^]^ These properties are crucial for the construction of a large‐scale mechanoreceptor array.

Based on the spiking behaviors of the proposed mechanoreceptor array, utilized for detecting various force levels, ten types of touch gestures were characterized. The arrayed mechanoreceptor cells could discern spatiotemporal information by simultaneously detecting both the trajectory and force applied. This capability is unattainable with a single mechanoreceptor unit. Finally, it was confirmed that the ten touch gestures could be accurately classified based on the data generated by the proposed mechanoreceptor array with the aid of SNN simulations.

## 2 Results and Discussion

### 2.1 Characteristics of the Triboelectric Nanogenerator (TENG) and Biristor

**Figure 2:**
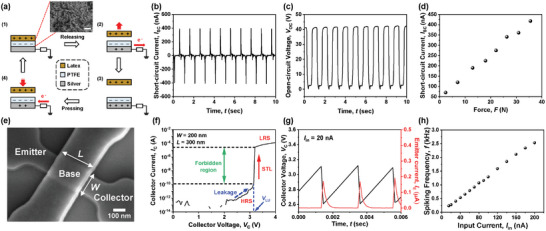
Measured characteristics from a TENG and a biristor. a) Operational mechanism of the TENG. Inset is a SEM image of the nanostructures formed on the PTFE surface. The scale bar is 1 µm. b) Measured *I*_SC_ of the TENG. c) *V*_OC_ of the TENG. d) *I*_SC_ as a function of the applied force. e) SEM image of a biristor with *L* equal to 300 nm and *W* equal to 200 nm. The scale bar is 100 nm. f) Output characteristics (*I*_C_ – *V*_C_) of the biristor. The forbidden region represents a transition range of *I*_C_ between the HRS and LRS. g) Wedge‐shaped *V*_C_ spiking characteristic of the biristor and spike‐shaped *I*_E_ characteristic of the biristor. *V*_C_ and *I*_E_ were simultaneously measured. h) Extracted *f* as a function of *I*_in_.

A serially connected TENG and biristor is the basic component of the proposed artificial mechanoreceptor. The TENG was designed to operate in single‐electrode mode, as shown in **Figure** **2**a.^[^ ^32^ ^]^ Polytetrafluoroethylene (PTFE) and silver were employed as the triboelectric surface and the electrode, respectively. Both length and width are 1 cm. Physical surface modification was applied to the PTFE, creating nanostructures to enhance the electrical output and sensitivity to external pressure.^[^ ^33^, ^34^, ^35^ ^]^ The electrical output was characterized while using a latex as the external contact material. During iterative contact‐separation between the latex and PTFE, alternating current (AC) is generated by the TENG. Through contact electrification, the latex becomes positively charged, while the PTFE becomes negatively charged according to the triboelectric series (Stage 1). When the upper latex is separated from the lower PTFE, electrons flow from the silver to the ground (Stage 2). In other words, positive charges are induced on the silver to compensate for the negative charges on the PTFE. Subsequently, electrons cease to flow once the latex moves a sufficient distance away (Stage 3). Next, as the upper latex moves closer to the lower PTFE, electrons flow from the ground to the silver (Stage 4). Figure 2b,c show the measured short‐circuit current (*I*_SC_) and open‐circuit voltage (*V*_OC_) of the TENG, respectively. Downward force of 35.5 N corresponding to pressure of 51.5 psi was applied by an electrodynamic shaker with the operating frequency of 1 Hz. The measured *I*_SC_ and *V*_OC_ values were 418 nA and 47.1 V, respectively. To verify the force sensitivity of the TENG, *I*_SC_ and *V*_OC_ were measured while varying the magnitude of the force. As shown in Figure 2d, the *I*_SC_ value measured from the TENG increased linearly as the downward force applied to the TENG was increased. This linearity is crucial for it to function effectively as a touch sensor.^[^ ^36^, ^37^ ^]^ It is also important to investigate endurance and stability of the TENG. The endurance and stability tests were conducted, as shown in Figure S2 (Supporting Information). There was no obvious degradation in *V*_OC_ measured from the TENG even after 10 000 contact‐separation cycles. Furthermore, the *V*_OC_ did not decrease significantly over five days, with the dates changed each time. These results indicate the good robustness and stability of the TENG as a touch sensor.

A few works have reported a biristor utilized as an artificial neuron due to its inherent spiking behavior.^[^ ^38^, ^39^, ^40^ ^]^ The nominal sizes of the biristor were a base length (*L*) of 300 nm and a base width of (*W*) of 200 nm. Figure 2e displays a scanning electron microscope (SEM) image of the biristor. It is composed of an n^+^ emitter (E), a p‐type floating base (B), and an n^+^ collector (C)—a structure essentially identical to a metal‐oxide‐semiconductor field‐effect transistor (MOSFET) with a floating body but without a gate electrode. In other words, the gate physically existed but it was not electrically utilized. The fabrication details of the biristor are provided in the Experimental section and in Figure S3 (Supporting Information). The measured output characteristic of the collector current (*I*_C_) versus the collector voltage (*V*_C_) is plotted in Figure 2f. *I*_C_ increased abruptly at a critical voltage, defined as the latch‐up voltage (*V*_LU_) by single transistor latch (STL).^[^ ^41^, ^42^ ^]^ The STL caused the high‐resistance state (HRS) of 20.4 GΩ to decrease rapidly to a low‐resistance state (LRS) of 115.4 kΩ at *V*_LU_. These two resistance states with a significant difference resulted in two bi‐stable states of the biristor. Thus, a forbidden region existed between the HRS and the LRS. Referring to the energy band diagrams and circuit models depicted in Figure S4 (Supporting Information), the mechanism of the STL and leaky integrate‐and‐fire (LIF) operation can be explained. When input current (*I*_in_) was applied to C, *V*_C_ increased linearly because the biristor is an HRS before the occurrence of the STL. This charging process corresponds to the “integrate” characteristic of neuronal operation. Subsequently, *V*_C_ abruptly decreased *vi*a the STL, driving the transition from the HRS to the LRS when *V*_C_ surpassed *V*_LU_. At the moment of the STL, the stored charges when in the HRS suddenly escaped to E. This discharge corresponds to the “fire” characteristic of neuronal operation. Subsequently, the biristor automatically returned to the HRS because the stored holes in B escaped out. On the other hand, the incremental leakage current before *V*_C_ reached *V*_LU_ in Figure 2f played a dominant role in creating a “leaky” characteristic during neuronal operation.^[^ ^43^ ^]^ Such iterative LIF operations, represented by wedge‐shaped *V*_C_ spikes, are sustained while *I*_in_ is fed to **C**, as exhibited in Figure 2g. Additionally, the abovementioned firing operation of the biristor was observed at E by characterizing the emitter current (*I*_E_), which is plotted in Figure 2g. Here, *I*_E_ showed an abrupt increase at the moment of the firing; therefore, a spike‐shaped signal was also observed. Also, as shown in Figure 2h, the spiking frequency (*f*) extracted from the biristor increased linearly as *I*_in_ was increased because more rapid integration of charges is induced with the higher *I*_in_. This is another typical neuronal characteristic.

### 2.2 Hardware Implementation of the Artificial Mechanoreceptor Array

**Figure 3:**
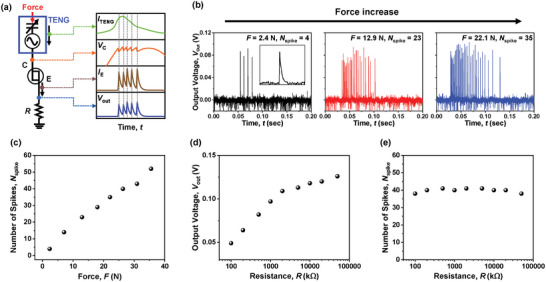
Measured characteristics from a single mechanoreceptor cell. a) Circuit diagram of the proposed mechanoreceptor cell. When force is applied to the TENG, *I*_TENG_ generated from the TENG is fed to the biristor as input current (*I*_in_). Then, outputs with a train of serial spikes are produced in the form of *V*_C_, *I*_E_, and *V*_out_. b) Different numbers of spikes according to the applied force. c) Extracted *N*_spike_ as a function of the applied force. d) *V*_out_ as a function of *R* at a fixed force of 26.3 N. e) Extracted *N*_spike_ as a function of *R* at a fixed force of 26.3 N. *N*_spike_ are not affected significantly by a change of *R*.

The proposed artificial mechanoreceptor cell was made by combining a TENG as the tactile sensing part and a biristor as the spike‐generating part, as plotted in **Figure** **3**a. These two components were serially connected. When external force is applied, the output current (*I*_TENG_) generated from the TENG flows into C of the biristor as *I*_in_, which enables LIF operation of the biristor. *I*_TENG_ integrates the positive charges at C and firing occurs when *V*_C_ reaches *V*_LU_. With a resistor (*R*) connected in series with E of the biristor, the abovementioned *I*_E_ is delivered to *R*, after which output voltage (*V*_out_) of *I*_E_⋅*R* is induced according to Ohm's law. Therefore, a single touch on the TENG produces *I*_TENG_ to trigger iterative LIF operations quantified as *V*_C_, *I*_E_, *V*_out_, and *f*, as plotted in Figure 3a. To check whether the value of *I*_TENG_ from the TENG is sufficient to drive the LIF operation of the biristor, *I*_TENG_ generated from the TENG was measured for various load resistance values (*R*_load_). As *R*_load_ increases in Figure S5 (Supporting Information), *I*_TENG_ decreases owing to the slower speed of the charge transfer between the electrode of the TENG and the ground. The measured *I*_TENG_ with an *R*_load_ value of 20 GΩ, which corresponds to the HRS of the biristor, was within the abovementioned forbidden region in the output characteristic of the biristor. Accordingly, *I*_TENG_ is high enough to trigger the LIF operation, enabling the biristor to function as a processing unit without a power supply.^[^ ^38^ ^]^

With the prepared artificial mechanoreceptor, *V*_out_ was measured through a data acquisition board (DAQ) while changing the external force. Here, the applied force was varied in a range from 2 N (corresponding to 2.9 psi) to 60 N (corresponding to 87.0 psi), reflecting the usual force when using a keyboard.^[^ ^44^, ^45^, ^46^ ^]^ As shown in Figure 3b,c, the number of spikes by a single touch (*N*_spike_) increased proportionally as the applied force was increased, implying that the artificial mechanoreceptor can detect force levels and encode them into *N*_spike_. In addition, *N*_spike_ increased linearly as the contact area of the TENG (*A*_TENG_) was enlarged. This *A*_TENG_, depicted as a scalable parameter in Figure S6 (Supporting Information), has been reduced to the size of a pen tip. Such a small‐sized TENG can still trigger the STL and drive the encoding process. If a size of the biristor is reduced further, *V*_LU_ tends to be smaller and the required *I*_in_ (= *I*_TENG_) becomes smaller. Accordingly, *A*_TENG_ to govern *I*_TENG_ can also be decreased further.^[^ ^30^ ^]^ On the other hand, it was confirmed that *V*_out_ could be effectively modulated by varying *R* while *N*_spike_ remained unchanged (Figure 3d,e), as *N*_spike_ is determined by the number of latch cycles, which only depends on *V*_LU_.

**Figure 4:**
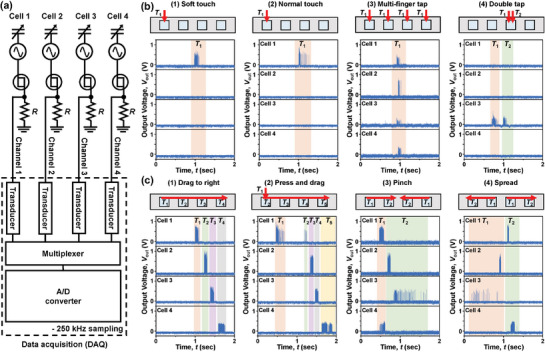
Hardware configuration of the mechanoreceptor with a 1 × 4 array and measured output voltages for the different gestures. a) Multi‐channel measurement setup to obtain the spiking characteristics acquired through the DAQ board. b) Corresponding *V*_out_ signals with each different trajectory under various tapping‐type gestures. c) Corresponding *V*_out_ signals with each different trajectory under various dragging‐type gestures.

To demonstrate the detection ability by tracing the spatiotemporal and dynamic mechanical pressure, four TENGs were arrayed in a single row with an interval of 1 cm for an artificial mechanoreceptor module, as illustrated in Figure S7 (Supporting Information). Each TENG was serially connected with each corresponding biristor and resistor. **Figure** **4**a shows the measurement setup used to characterize the signals from the mechanoreceptor array. The four mechanoreceptor combinations are denoted as Cell 1, Cell 2, Cell 3, and Cell 4. All the mechanoreceptor cells were then connected to the DAQ board, where *V*_out_ was acquired in real‐time. To validate the gesture recognition capability of the proposed array, two categories of ten gestures, tapping‐type and dragging‐type gestures, were applied. The four tapping types include a soft touch, a normal touch, a multi‐finger tap, and a double tap. The six dragging types consist of a drag to the right, a drag to the left, a press and drag, a pinch, a spread, and a two‐finger drag. Figure 4b shows the four trajectories with their corresponding *V*_out_ values for the tapping‐type gestures. Figure 4c exhibits the four paths with their corresponding *V*_out_ values for the dragging‐type gestures. Figure S8 (Supporting Information) displays the other two trajectories with their corresponding *V*_out_ values for the remaining dragging‐type gestures. Herein, the temporal sequence of touch events is labeled *T*_1_, *T*_2_, …, *T*_n_, respectively. For example, when *T*_1_ is allocated to all cells (Figure 4b (3)), it implies that these cells are pressed simultaneously at *T*_1_. If *T*_1_ and *T*_2_ are designated for a single cell (Figure 4b (4)), it indicates that the cell was touched at *T*_1_ and *T*_2_, respectively, twice in total. Different types of complicated gestures were defined in combination with *T*_i_ (1 ≤ i ≤ n), as illustrated in Figure 4b and Figure S8 (Supporting Information).

Because the proposed mechanoreceptor encodes the force level into spikes, the resultant *V*_out_ from the mechanoreceptor array reflects both the force level and the response time. Thus, the applied finger trajectory is well matched to the acquired *V*_out_ signals. For instance, *N*_spike_ under a normal touch exceeds that under a soft touch when comparing Figure 4b(1,2). When four TENGs were tapped simultaneously, spikes were observed in all four cells, as shown in Figure 4b (3). Furthermore, the groups of spikes were temporally separated when the same TENG was consecutively touched twice within a short time interval of 0.3 sec, as shown in Figure 4b (4). Similarly, for the dragging‐type gestures, each unique spike pattern in *V*_out_ corresponded to each touched TENG, as shown in Figure 4c and Figure S8 (Supporting Information). In contrast, when a TENG was untouched or very weakly touched, it could not deliver *I*_TENG_ to a connected biristor. Consequently, no spikes were observed in that cell. It should be noted that all spikes were spatially identified for all ten types of gestures, in addition to temporal classification.

### 2.3 Spike‐Based Touch Gesture Recognition

**Figure 5:**
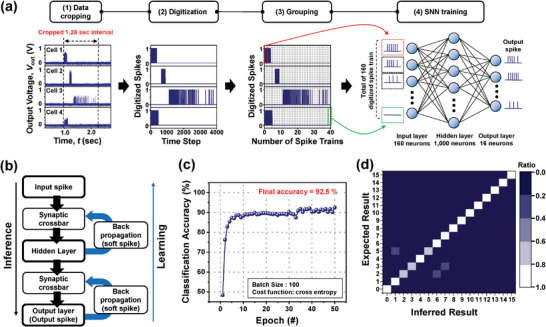
SNN simulation for the recognition of touch gestures. a) Procedure of data processing in the SNN configuration. b) Sequential flow for inference and learning through SNN training. c) Simulated classification accuracy per epoch. d) Confusion matrix of the test result.

In the section above, we verified the one‐to‐one mapping between the applied gestures in the form of force and the unique output patterns in the form of *V*_out_ spikes using the hardware of the mechanoreceptor array. Herein, to evaluate the classification ability of the touch gestures shown in Figure 4 and Figure S8 (Supporting Information), semi‐empirical SNN simulations reflecting the data acquired by the hardware were conducted. This was achieved by modifying the well‐developed open‐source SNN code produced by Duan et al.^[^ ^47^ ^]^ Table S1 (Supporting Information) summarizes the 16 gestures used for the classification. A serial number from ‘0′ to ‘15′ is assigned to each gesture in the column. Note that there are ten gestures and 16 classes. The normal touch and soft touch gestures were divided into four sub‐classes each based on the number of touched cells. **Figure** **5**a shows the overall data processing procedure for the simulation. The configuration of the SNN consists of three layers containing 160 neurons for the input layer, 1000 neurons for a hidden layer, and 16 neurons for the output layer corresponding to each of the 16 gesture types. Prior to feeding input data into the input neurons, there were four sequential procedures for each cell. In (1), input data were cropped at intervals of 1.28 sec. This cropping started from the time when any of the four cells initially fired a spike. Next, the time interval of 1.28 sec was discretized into sub‐time steps of 4000, making each sub‐time step 320 µsec. In (2), the continuous values of *V*_out_ were digitized to 0 or 1 depending on the presence of a spike at each sub‐time step. Consequently, there were 4000 digitized spikes for the time interval of 1.28 sec. In (3), the 4000 digitized spikes were grouped into 40 trains. For the 1 × 4 array, there were 160 digitized spike trains. In (4), each train of 160 was fed into each of the 160 input neurons. *V*_LU_ of the neurons at the hidden layer and output layer was set to 3 V, which is comparable to the measured *V*_LU_ from a fabricated biristor. It was designed from the perspective that input layer neurons (mechanoreceptor array), hidden layer neurons, output layer neurons and synapses between each layer can be integrated using CMOS process technology.^[^ ^30^ ^]^

To train the SNN, we conducted each gesture repeatedly and the collected data were then used for feeding to the input neurons. Because the number of actually measured input data instances was not sufficient for SNN training, additional artificial data points were generated based on the original data. Details of the method used to generate the artificial data are summarized in Note S1 and Figure S9 and S10 (Supporting Information). As a result, 1700 data points, augmented from 145 measured data points, were used for the SNN training. The procedure for SNN training is depicted in Figure 5b. With regard to inference, each digitized spike is weighted by the synaptic crossbar and passed to the hidden layer neurons. When the weighted data are summed at the hidden layer neurons, the *V*_C_ values of the hidden layer neurons are accordingly modulated. Each neuron in the hidden layer performs the LIF operation, as that in the input layer does, because it is also a biristor used as an artificial neuron. Thus, when *V*_C_ reaches *V*_LU_, it generates the spikes, after which *V*_C_ is abruptly dropped again;, that is, it returns to the HRS state. Afterward, the spikes generated in the hidden layer neurons are transmitted to the output layer neurons through the synaptic crossbar with weights. The output layer neurons then generate the output spikes only when *V*_C_ reaches *V*_LU_. Finally, the number of spikes at each output neuron was counted, and the complex gestures were classified into a class based on which corresponding output neuron fired the most. To conduct the training, the inference result for each data pattern was vectorized into a vector with firing rates of the output neurons as elements, and the inference result vector was then fed into the softmax function. Subsequently, the loss for each data pattern was calculated using the cross‐entropy function, comparing the inference result vector to the one‐hot encoded target vector where only the element with an index identical to the correct class number (label) has a value of 1. For more effective training, the mini‐batch strategy was employed in this study with a batch size set to 100. After testing each mini‐batch, the overall loss for that batch was calculated, and gradients were derived using the backpropagation algorithm. Due to the infinite gradient of the neuron's spike function, a soft sigmoid function was utilized during the backpropagation process, similar to earlier works.^[^ ^21^, ^47^ ^]^ Afterward, weight updates were performed based on the gradients using the Adam optimizer, and the updated weight values were quantized to a finite 7‐bit level to facilitate future integration with synaptic devices.

After 50 epochs of training, high classification accuracy of 92.5% was achieved when the SNN was tested with the training set (Figure 5c). This was also confirmed in the confusion matrix, as displayed in Figure 5d and Figure S11 (Supporting Information). Because this matrix map represents the correlation strength between the inferred result and the expected result, the inferred result was deemed reliable, as indicated by the high values along the diagonal direction. These results provide evidence that the proposed mechanoreceptor array has high potential for event‐driven gesture recognition through SNN training. It is also important to note that not only gestures with distinctly different patterns but also soft touches and normal touches with very similar patterns can be classified well because the mechanoreceptor proposed in this study has a high force resolution in both the spatial and temporal domains.

## 3 Conclusion

In this work, we demonstrated a self‐powered mechanoreceptor array for spike‐based neuromorphic computing. A single mechanoreceptor cell consists of a triboelectric nanogenerator (TENG) and a bi‐stable resistor (biristor). A TENG was used as an energy harvester to power mechanoreceptor cells and acted as a force sensor capable of transducing applied physical force into an electrical signal in the form of current and voltage. A biristor was utilized as an artificial neuron. The current generated from the TENG was fed to the collector of the biristor when the TENG sensed external force. Subsequently, the biristor performed repetitive integrate‐and‐fire operations and produced voltage signals in the form of spikes. Thus, the proposed artificial mechanoreceptor spontaneously generated spike signals even without an external power supply. Here, the applied force was characterized by the number of spikes. Given that this artificial mechanoreceptor encodes information with spike signals, it can adopt a spike‐based neuromorphic computing architecture rather than the power‐hungry von‐Neumann computing architecture. By configuring the mechanoreceptor array, the system can perceive complicated spatiotemporal patterns. Four channels of the data acquisition (DAQ) board obtain spiking output voltages from four mechanoreceptor cells. The position of the pressed TENG and its accompanying data acquisition time deliver spatial and temporal information, respectively. After characterizing the spike signals from the mechanoreceptor array, complex touch gestures were classified with the software‐based spiking neural network (SNN) by reflecting the measured spike characteristics. From a long‐term perspective, the proposed self‐powered mechanoreceptor array can be applied in various fields, such as robotics, prosthetics, diagnosis, and human‐machine interfaces. Specifically, with the high resolution of the mechanoreceptor array in terms of the force level, space, and time, an arbitrary calligraphic pattern can be identified to certify one's genuine signature.

## 4 Experimental Section

### Fabrication of the Biristor

The biristor was fabricated on an 8‐inch p‐type (100) silicon‐on‐insulator wafer. A silicon channel was 50 nm. An active (channel) area was patterned by photolithography and etching. After the deposition of a gate hard mask to function as a subsequent implantation stopper, it was patterned by another photolithography and etching. Arsenic implantation and subsequent rapid thermal annealing (RTA) using a self‐aligned doping technique were applied to create the emitter and collector regions, where n^+^ doping was conducted, except in the shaded p‐type area covered by the gate hard mask. Therefore, the biristor has the configuration of an emitter (n^+^), a base (p), and a collector (n^+^). The overall process flow, showing cross‐sectional views in a stepwise manner, is graphically illustrated in Figure S3 (Supporting Information).

### Fabrication of the TENG

The TENG consists of three layers consisting of one external stimulus layer of latex and two internal structural layers of PTFE and silver, as illustrated in Figure S7 (Supporting Information). The PTFE and latex act as triboelectric layers, while silver serves as a single electrode of the TENG. To improve the sensitivity of the TENG to subtle applied force, nanostructures were fabricated on the PTFE to enhance the electrical output performance by enlarging the contact area. In detail, after cleaning the PTFE film with isopropyl alcohol, 4 nm of gold (Au) was deposited on the PTFE film via e‐beam evaporation to form nanoscale island patterns, which serve as etching stoppers. The PTFE uncovered by the Au masks was then partially etched by Ar, CF_4_, and O_2_ plasma using a polymer etcher (VL‐ICP, Unaxis) with RF power of 400 W and a bias power of 100 W. The flow rates of the Ar, CF_4_ and O_2_ used here were 15, 40, and 10 sccm, respectively. As a result, protruding nanostructures were fabricated on the PTFE film with a size of 1 cm × 1 cm, which served as a triboelectric layer. This was then mounted over a silver electrode patterned on a printed circuit board. As the counter triboelectric layer to the PTFE, latex was used.

### Characterization and Measurement

A field‐emission scanning electron microscope (JSM‐IT800, JEOL) was used to obtain a SEM image of the biristor and the PTFE surface morphology. To apply force to the TENG, an electrodynamic shaker (LW‐140‐110, LabWorks) connected to a function generator (33120A, HP) was utilized. The electrical properties of the TENG were characterized using an electrometer (6514, Keithley). The electrical properties of the biristor were characterized using a semiconductor parameter analyzer (B1500A, Keysight). A DAQ board (USB‐6225, NI) was used to measure the electrical properties of each mechanoreceptor cell.

## Conflict of Interest

The authors declare no conflict of interest.

## Supporting information

Supporting Information

## Data Availability

The data that support the findings of this study are available from the corresponding author upon reasonable request.
