# Supplementary material for: Spike‐Based Neuromorphic Hardware for Dynamic Tactile Perception with a Self‐Powered Mechanoreceptor Array
Source: Adv Sci (Weinh). 2024 Jul 9;11(34):2402175. doi: 10.1002/advs.202402175 (PMC11425894; doi:10.1002/advs.202402175)
Supplement: Supplementary file 1 — Supporting Information [file ADVS-11-2402175-s001.docx]

**Supporting Information**

**Spike-Based Neuromorphic Hardware for Dynamic Tactile Perception with a Self-Powered Mechanoreceptor Array**

*Sang-Won Lee* †*, Seong-Yun Yun* †*, Joon-Kyu Han* *, Young-Hoon Nho* *, Seung-Bae Jeon* ^*^*, and Yang-Kyu Choi* *

S.-W. Lee, S.-Y. Yun, Y.-K. Choi

School of Electrical Engineering, Korea Advanced Institute of Science and Technology (KAIST), 291 Daehak-ro, Yuseong-gu, Daejeon 34141, Republic of Korea

J.-K. Han

System Semiconductor Engineering and Department of Electronic Engineering, Sogang University, 35 Baekbeom-ro, Mapo-gu, Seoul 04107, Republic of Korea

Y.-H. Nho

Department of Neurosurgery, University of Pennsylvania, Philadelphia, PA 19104, USA

S.-B. Jeon

Department of Electronic Engineering, Hanbat National University, 125 Dongseo-daero, Yuseong-gu, Daejeon 34158, Republic of Korea

†*These authors equally contributed to this work.*

E-mail: ykchoi@ee.kaist.ac.kr and sbjeon@hanbat.ac.kr

**Supplementary Figures**

**1.** **Comparison of tactile system between conventional von Neumann and spike-based neuromorphic architecture**

**Figure S1.** Comparison of (a) artificial tactile system with conventional von Neumann architecture and (b) that with spike-based neuromorphic architecture. The proposed mechanoreceptor array is based on spike-based neuromorphic architecture, *i.e.*, spiking neural network (SNN). Due to absence of two bottlenecks, limits of high cost and power consumption in hardware can be overcome by adopting a SNN in the artificial tactile system.

**2. Robustness and stability of TENG**

**Figure S2.** Endurance and stability test of the TENG. (a) Measured *V*_OC_ from the TENG up to 10,000 cycles of contact-separation. (b) Close-up view of the dashed box in (a). (c) Measured *V*_OC_ from the identical TENG for five different days.

**3.** **Fabrication procedure of biristor**

**Figure S3. Cross-sectional schematics at each step for the fabrication of a biristor.** (a) A p-type (100) silicon-on-insulator (SOI) wafer with the buried oxide (BOX) was prepared as a starting substrate. A p-type top silicon was thinned down using sacrificial oxidation and its removal. An active channel area was delineated by photolithography and plasma etching. (b)

Hard mask materials composed of a gate oxide and a poly-Si gate, which can function as a dummy gate with physical existence but no electrical functionality, were deposited as an implantation stopper before the subsequent n^+^ implantation. (c) Hard mask was patterned by another photolithography and plasma etching. (d) Ion implantation with arsenic and subsequent rapid thermal annealing (RTA) were applied.

**4.** **Mechanism of LIF operation in biristor**

**Figure S4.** Mechanism of LIF operation in a biristor. (a) Symbolic representation of the biristor in (1), its cross-sectional schematic in (2), and corresponding energy band diagram along the *A*-*A*’ direction in (3). (b) Step-by-step energy band diagrams to illustrate the mechanism of the transition from HRS to LRS. When *V*_C_ is applied to the **C**, the energy level at the collector junction is lowered and leakage current composed of electrons starts to flow at the HRS in (1). When the injected electrons are accelerated by the increased *V*_C_, impact ionization (II) is triggered to create additional electron and hole pairs at the collector junction. The generated holes are stored in the floating **B**, lowering the potential barrier between the **E** and **B**. Simultaneously, the generated electrons flow out toward the collector in (2). Consequently, as more electrons overcome this reduced potential barrier, they enhance a rate of II, causing the potential barrier to decrease further. This serial process creates a positive feedback loop. When the *V*_C_ reaches a critical value of *V*_LU_, the biristor abruptly changes its resistance state from HRS to LRS in (3). (c) Circuit diagram to describe LIF operation of biristor neuron. When *I*_in_ is fed to the **C**, positive charges are integrated at a parasitic capacitor (*C*_para_), which is connected to the biristor in parallel. Then, the induced voltage (*V*_cap_) at the *C*_para_, which is equivalent to *V*_C_, increases in (1). This process corresponds to ‘integrate’ operation of the biristor neuron. It is worth noting that *C*_para_ includes probing pad capacitance, cable capacitance, and equipment (parameter analyzer) capacitance. When the *V*_cap_ reaches *V*_LU_, the abrupt transition from HRS to LRS is occurred, as shown in the energy band diagram in (b). As a result, positive charges flow out to the ground through the biristor in (2). Due to the discharging of positive charges, *V*_cap_ drops suddenly. This process indicates ‘fire’ operation of the biristor neuron. Again, the biristor automatically returns to HRS as a resting process with no demand of extra circuits. The biristor iteratively integrates another positive charge for a next round firing as long as the *I*_in_ is supplied to the biristor in (3). This continuous LIF operations result in generating iterative spike signals with *f*.

**5.** **Output characteristics of TENG for different *R*_load_**

**Figure S5.** Output characteristics of the TENG for different *R*_load_. (a) Equivalent circuit diagram to characterize *I*_TENG_ for various *R*_load_ **­**from 2 kΩ to 50 GΩ. (b) Measured *I*_TENG_ from the TENG versus time at *R*_load_ of 20 GΩ, corresponding to the resistance of the biristor in HRS, where *I*_TENG_ can be delivered to the biristor.

**6.** **Linear relation between extracted *N*_spike_ and manufactured contact area of the TENG**

**Figure S6.** Extracted *N*_spike_ as a function of a contact area of the TENG (*A*_TENG_) at the force of 35.5 N. There is linear relation between the *N*_spike_ and *A*_TENG_. By extrapolation, the minimum *A*_TENG_ to generate spikes is expected to be 0.07 cm^2^.

**7.** **Fabricated TENGs on PCB for measurement of mechanoreceptor array**


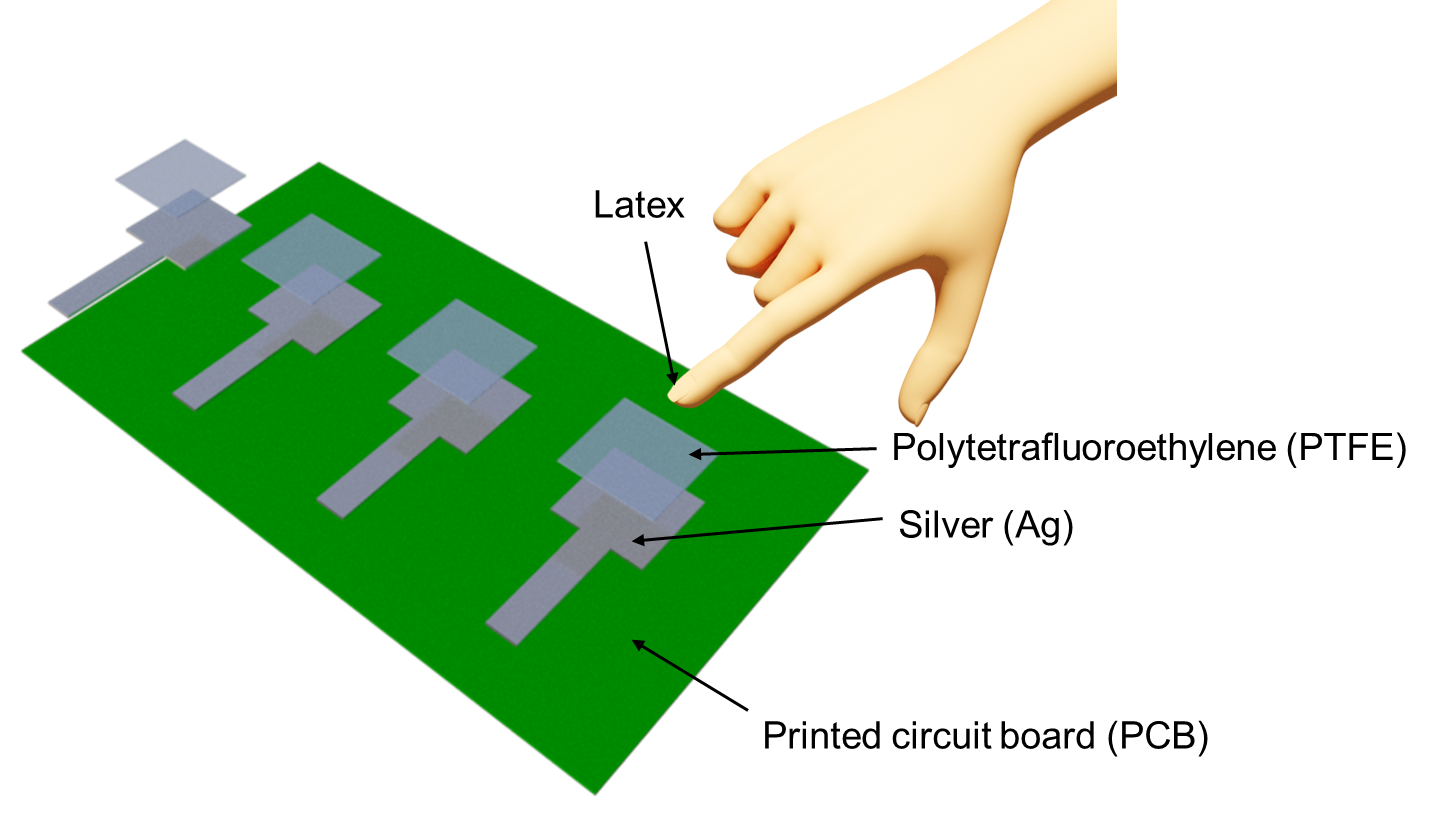


**Figure S7.** Schematic illustration of 1 × 4 TENG array with silver patterns on a PCB for measurement. A triboelectric PTFE film with protruded nanostructures was formed and positioned over the silver electrode. Here, to create the protruded nanostructures on the PTFE film, a polymer etcher with Ar, CF_4_, and O_2_ plasma was used. On the other hand, latex was selected as a counter triboelectric layer to the PTFE film.

**8.** **Corresponding output voltage with different trajectories under dragging-type gestures**

**Figure S8.** Corresponding *V*_out_ signals with each own trajectory for ‘drag to left’ and ‘two fingers drag’.

**9.** **Data augmentation for software simulations**

**Supplementary Note 1:** Here, detailed procedure of data augmentation is explained using the ‘drag to right’ gesture as an example. We repeatedly conducted the ‘drag to right’ gesture n-times. Afterwards, parameters such as intermittent time (*t*_int_) and duration time (Δ*t*) were extracted from the original dataset (Figure S9a). The *t*_int_ implies the elapsed time after the first cell is activated. When running n-times, there are *t*_int,1_, *t*_int,2_, …, and *t*_int,n_. Among them, *t*_int,min_ represents the minimum value and *t*_int,max_ expresses the maximum value. The duration time (Δ*t*) refers to the sustained period of spike activation. Similar to the above, there are Δ*t*_1_, Δ*t*_2_, …, and Δ*t*_n_ when running n-times. Among them, Δ*t*_min_ represents the minimum value and Δ*t*_max_ denotes the maximum value. Then, artificial parameters represented as *t*_int,art_ and Δ*t*_art_ were randomly set within the ranges of *t*_int,min_ to *t*_int,max_ and Δ*t*_min_ to Δ*t*_max_. As an example, *t*_int,art_ and Δ*t*_art_ were generated for the ‘drag to right’ gesture. Schematics for how to extract parameters from the measured data and how to set parameters for an artificial data are shown in Figure S9b and c.

After setting the *t*_int,art_ and Δ*t*_art,_ the random extraction of a digitized spike (0 or 1) was conducted from the originally measured data at every sub-time step during the Δ*t*_art_, and therefore, artificial generated data could be attained (Figure S9d). Detailed procedure of the random extraction of spikes from the measured data to generate artificial data is displayed in Figure S10. To illustrate the process, generation of artificial data for Cell 4 was used as an example, considering data generation based on the ‘parameter setting’ shown in Figure S9c. For the convenient explanation, the number of sub-time steps created by the Δ*t*_art_ in Cell 4 is assumed to be 536, and the index from ‘0’ to ‘535’ was assigned to each sub-time step. To generate artificial spike (0 or 1) in each sub-time step, the following steps are taken: (1) random selection of measured data; (2) insertion of a spike (0 or 1) at the sub-time step with the same index of the selected measured data into the same index of artificial data. This procedure of (1) and (2) is conducted for every sub-time step within Δ*t*_art._ For example, if the measured data #1 is randomly selected for the sub-time step index ‘0’ of the artificial data, the spike (0 or 1) occurring at the 1^st^ sub-time step of the measured data #1 is pasted to the sub-time step index ‘0’ of the artificial data. In the same sense, if the measured data #5 is selected for the sub-time step index ‘535’ of the artificial data, the spike (0 or 1) occurring at the 536^th^ sub-time step of the measured data #5 is pasted to the sub-time step ‘535’ of the artificial data. If the index of the sub-time step of the artificial data is larger than the total number of the sub-time steps in Δ*t* of the selected measured data, the ‘0’ was filled into that sub-time step.

**Figure S9. Data augmentation procedure for the software simulations.** Here, the gesture of ‘drag to right’ was chosen as an example. (a) Overall procedure of parameter extraction from the measure data and parameter setting for an artificial data. (b) Detailed procedure of ‘parameter extraction’ with digitized spikes. (c) Detailed procedure of ‘parameter setting’ for an artificial data. (d) Example of the artificially generated data by data augmentation.

**Figure S10. Detailed procedure of the spike extraction from the measured data to generate artificial data.**

**10. Detailed Confusion matrix with the values of the ratio**


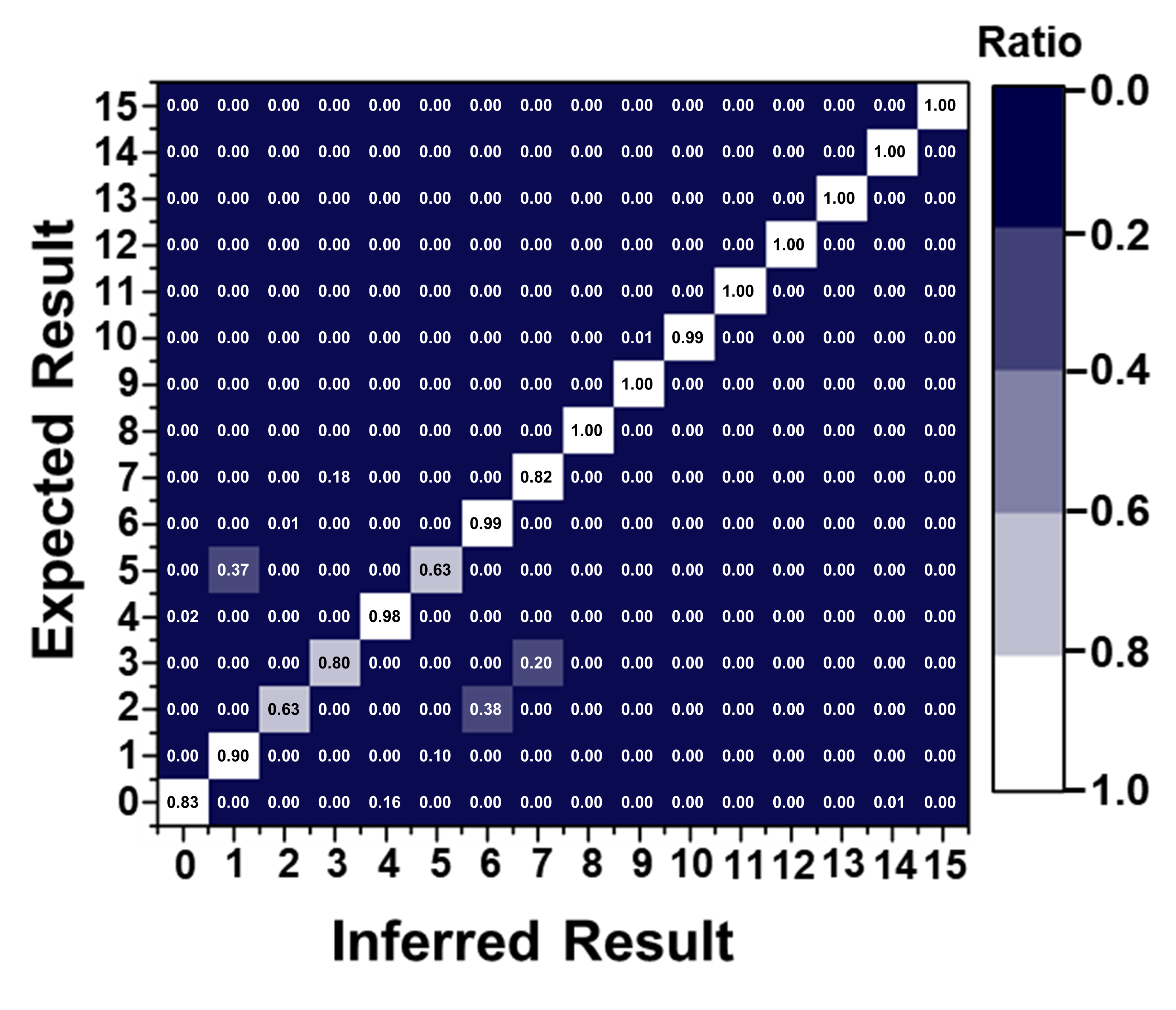


**Figure S11. Detailed confusion matrix with the values of the ratio.**

**Supplementary Table**

**1. Summarized table of the classified input gestures.**

| **Class No.** | **Gesture** |
| --- | --- |
| **1** | **Cell 1 soft touch** |
| **2** | **Cell 2 soft touch** |
| **3** | **Cell 3 soft touch** |
| **4** | **Cell 4 soft touch** |
| **5** | **Cell 1 normal touch** |
| **6** | **Cell 2 normal touch** |
| **7** | **Cell 3 normal touch** |
| **8** | **Cell 4 normal touch** |
| **9** | **Drag to right** |
| **10** | **Drag to left** |
| **11** | **Pinch** |
| **12** | **Spread** |
| **13** | **Press and drag** |
| **14** | **Multi-finger tap** |
| **15** | **Double tap** |
| **16** | **Two-finger drag** |
